# P259: What is the impact of hospital acquired bacteraemia in sub-Saharan Africa?

**DOI:** 10.1186/2047-2994-2-S1-P259

**Published:** 2013-06-20

**Authors:** A Aiken

**Affiliations:** 1 Department of Infectious Diease Epidemiology, London School of Hygiene and Tropical Medicine, London, UK

## Introduction

The impacts associated with hospital-acquired infections in developing countries are poorly understood, which is largely due to a lack of reliable primary data [1].

## Objectives

Following publication of a major study of paediatric nosocomial bacteraemia in a single hospital in Kenya [2], we aim to make a preliminary estimate of the mortality burden associated with this disease in sub-Saharan Africa.

## Methods

Using data on hospital admissions, per admission risk of disease and associated mortality from Kilifi Hospital, Kenya in 2005 and combining these with contemporaneous regional population estimates, we can make a first estimate of the number of deaths attributable to hospital-acquired bacteraemia in African children.

## Results

Based on this approach, we suggest that approximately 20,000 deaths in children under 5yrs might have occurred in sub-Saharan Africa in 2005 due to hospital-acquired bacteraemia. However, due to the inherent insensitivity of blood cultures for detecting bacteraemia in children, this might be a substantial underestimate.

## Conclusion

Hospital-acquired bacteraemia in developing countries could have a substantial mortality burden and further work is needed to define the scale and preventability of this problem.

## Competing interests

None declared
